# Over-connected? A qualitative exploration of smartphone addiction among working adults in China

**DOI:** 10.1186/s12888-019-2170-z

**Published:** 2019-06-18

**Authors:** Li Li, Trisha T. C. Lin

**Affiliations:** 1 School of Journalism, Yunnan University, Baijia Road, Kunming, Yunnan Province People’s Republic of China 650000; 2 Department of Radio and Television, College of Communication, National Chengchi University, Taipei, Taiwan

**Keywords:** Smartphone addiction, Symptoms, Psychological factors, Working adults, China

## Abstract

**Background:**

Smartphones currently dominate people’s lives and interests due to their increased affordability and functionality. However, the negative aspects of smartphone use, such as smartphone addiction, have recently been brought up. This study utilized a qualitative approach to explore the symptoms of smartphone addiction among working adults in China and the psychological factors that affect such addiction.

**Methods:**

Semi-structured interviews, either face to face or via Skype (online), were conducted with 32 Chinese workers. The collected data were analyzed using a thematic analysis approach in Nvivo 10 software.

**Results:**

This study identified four typical symptoms of smartphone addiction, namely, withdrawal (e.g., experiencing negative feelings when having no access to smartphones), salience (e.g., constant checking and thinking about smartphones), conflict (e.g., smartphone use interferes with family and work life), and phantom phone signals (e.g., illusory perception of a phone vibrating or ringing). Conscientiousness, neuroticism, and extroversion increase the likelihood of smartphone addiction. Notably, this study found that conscientious workers are likely to develop smartphone addiction, a finding that is contrary to those of the majority of existing studies on technological addiction.

**Conclusions:**

This study revealed various smartphone addiction symptoms among young Chinese workers, and these include withdrawal, salience, conflicts, and phantom phone signals. Conscientious, neurotic, and extroverted employees are likely to exhibit these symptoms.

## Background

Smartphones dominate people’s lives and interests due to their increased affordability and functionality. However, the negative aspects of smartphone use, such as smartphone addiction, have recently been brought up. Existing reports from various countries have shown that an increasing number of individuals cannot live without their smartphones, and over half of these individuals never leave home without their smartphones [1, 2]. A large-scale market research based on monitoring data on 1.3 billion smartphones has revealed that over 176 million people worldwide can be considered “mobile addicts” [3].

Although extensive academic research has investigated smartphone addiction in the past few years, most of the studies used samples of adolescents and university students [4–6].An increasing number of market surveys and anecdotal articles have reported that workers have become addicted to their smartphones, and this addiction results in negative outcomes, such as impaired social relationships and decreased job performance [7, 8]. Thus, understanding smartphone addiction in this social group is crucial. By interviewing 32 full-time working adults in China, this study aims to answer two research questions. (1) What are the symptoms of smartphone addiction among working adults in China? (2) What psychological factors affect their smartphone addiction?

The results of this pioneering study on smartphone addiction among working adults can deepen our understanding of this condition among the members of this little-known social group. Moreover, this study can help in the development of a valid symptom-based instrument for measuring smartphone addiction among working adults, in addition to examining the psychological factors that affect such addiction. Investigating the symptoms of smartphone addiction among working people and the influencing psychological factors could provide Chinese companies with valuable knowledge for the development of effective strategies to curb this problem at the early stage.

### Smartphone addiction

Smartphone addiction developed from the introduction and extensive usage of smartphones in modern society. Many studies have examined smartphone addiction [4, 6, 9–12]. Given that behavioral addiction is associated with negative notions and outcomes, smartphone addiction [4, 10, 13], problematic smartphone use [14], and compulsive smartphone use [15, 16] have been used interchangeably to describe the maladaptive behaviors or psychological impairments caused by the excessive use of and over-reliance on smartphones. In this study, the conceptualization of smartphone addiction is congruent with technological addiction, which is a subset of behavioral addiction and defined as the excessive interaction between man and machine [16–18]. Griffiths argued that technological addiction develops when individuals become heavily reliant on devices to obtain psychological benefits, such reduced negative mood [19]. Increased usage of a specific technology to obtain desirable outcomes increases the likelihood of addiction [20]. Smartphones are a prominent source of addiction because they allow people to install various applications and perform numerous compelling activities (e.g., mobile Internet and mobile instant messaging) that are in line with their personal needs and interests [2]. When individuals obtain favorable outcomes from smartphone use, they become addicted. A recent study conducted by Lin et al. showed that individuals that use smartphones are more likely to develop device dependency compared with people who use traditional mobile phones [5].

### Addiction symptoms

Prior studies have identified signs exhibited by young smartphone addicts; examples of these signs include preoccupation with their phones while performing important tasks, anxiety and disorientation when their phones are not in close proximity, excessive phone use, manifestation of negative emotions when phone usage is reduced, inability to refrain from using smartphones in inappropriate situations, and productivity loss [2, 21]. Several researchers have developed different dimensions for smartphone addiction. For instance, Bian and Leung classified smartphone addiction symptoms into five dimensions, namely, inability to control cravings, preoccupation, feeling anxious and lost, disregard of harmful consequences, and productivity loss [4]. Lee et al. created a compulsive smartphone usage scale based on six groups of symptoms, as follows: withdrawal, loss of control, salience, life dysfunction, conflict, and compulsion/persistence [15]. Although numerous symptoms of smartphone addiction have been identified in existing literature, these symptoms were mainly obtained from quantitative survey studies that investigated adolescents or young students. Conducting quantitative survey research that tests the relationships between predetermined factors is insufficient to explore possible smartphone addiction symptoms. Furthermore, the smartphone addiction symptoms exhibited by adolescents and students may not be applicable to other age or social groups, such as working adults, because these groups may use smartphones differently. To fill these research gaps, the current study uses a qualitative interview method to explore the symptoms of smartphone addiction among young working adults.

This study utilizes Brown’s behavioral addiction criteria to analyze Chinese working adults’ smartphone addiction symptoms [22, 23]. Extant research has successfully applied Brown’s criteria to measure the symptoms of technological addiction, including mobile phone [18], computer [24], Internet [20], and online game [25] addictions. Brown’s criteria have been proven applicable in various technological addictions; thus, it should be suitable for exploring the symptoms of smartphone addiction among young working adults. Additionally, Brown’s well-established framework includes the most comprehensive and well-defined symptoms. According to Brown [22, 23], the symptoms of technological addiction can be measured from seven psychological and behavioral perspectives. *Withdrawal* refers to the negative feelings (e.g., anxiety, irritability, and impatience) that arise when a person is unable to perform a certain activity. *Salience* pertains to when an activity dominates a person’s thoughts and behavior to the point that he or she is unable to stop thinking about it. *Conflict* represents the performance of an activity that results in conflict with other people or activities. *Tolerance* arises when people engaging in an activity gradually spend increasing amounts of time to obtain positive feelings. *Relapse and reinstatement* pertain to the symptom in which a person resumes an activity with the same level of vigor after he/she has attempted to reduce it. *Euphoria* reflects a “buzz” or a “high” feeling that occurs during an activity. These addiction criteria can help us identify smartphone addiction symptoms from the interview data.

### Psychological factors

Prior research has shown that smartphone addiction is associated with various psychological factors. According to Park et al. [26], people’s psychological traits, such as innovativeness and locus of control, are linked to smartphone dependency. Park and Lee found that people with high shyness, loneliness, and depression scores and low self-esteem scores are likely to become addicted to their smartphones [2]. Lin et al. demonstrated that the level of sociability of smartphone users is positively related to their smartphone dependency [5]. However, all of these factors were mainly identified from adolescents or college students and may not be applicable to older working adults.

This study uses the Big Five personality traits as a guide to explore the psychological factors that affect young workers’ smartphone addiction [27]. The Big Five personality model is organized hierarchically from narrow to broad dispositions with extraversion, agreeableness, conscientiousness, neuroticism, and openness to experience [27, 28]. *Extraversion* refers to how deep and intense one’s interactions with others are. Extraverts, who are generally sociable, active, and talkative, have an energetic disposition toward the outside world. *Neuroticism* refers to emotional instability. Neurotic individuals often feel anxious, angry, and sad, and they are highly sensitive to others’ responses and relationship maintenance. The degree of cooperation, compliance, and modesty are associated with *agreeableness*. Agreeable people are amicable and friendly. Competence, achievement, self-discipline, and dutifulness are associated with *conscientiousness*. Conscientious individuals strive to achieve their goals and have control over their impulses. *Openness to experience* refers to the degree of a person’s intellectual curiosity, creativity, and preference for novelty. When individuals are open to experience, they are usually adventurous and willing to explore new things. These five personality traits that have been independently validated by different researchers capture most of the individual differences in human personalities [10, 29]. We believe that these traits can help understand the personality of Chinese workers and analyze the relationships between personality differences and smartphone addiction symptoms.

## Methods

The purpose of the current study is to investigate the symptoms of smartphone addiction among working adults in China and the psychological factors that affect such addiction. Semi-structured interviews were conducted from February to March 2015. Young workers with different professions and belonging to different gender groups were purposefully recruited and interviewed to obtain diverse perspectives. Thirty-two participants (17 males and 15 females) aged between 24 and 34 years (M = 28.09, SD = 2.79) were recruited through convenience and snowball sampling techniques. The participants worked in various industrial sectors in mainland China, and all of them regarded the smartphone as an indispensable tool in fulfilling their professional and social needs. With the exception of voice calls and SMS, they used mobile instant messages (e.g., WeChat) and mobile emails the most frequently. Although this study only interviewed 32 participants, the resulting data can reach data saturation for each theme because additional valuable information can be generated after interviewing more than 32 participants.

Interviews were conducted face to face or via Skype (online). They were based on a pre-established set of questions developed from related literature and research questions. The interview guide primarily covered three parts: (1) smartphone addiction symptoms, (2) psychological factors that affect addiction symptoms, and (3) demographics. Each interview was conducted for approximately 30 min, and a digital recording of each participant’s interview was created and stored for further analysis. For consistency, all interviews were conducted in Chinese by the same interviewer, who has been trained in qualitative research. Prior to the survey, approval was sought from the Institutional Review Board (IRB) of Nanyang Technological University. Furthermore, each interviewee participated voluntarily with proper consent.

A thematic analysis approach was utilized to analyze the data obtained from the interviews. First, audio recordings were transcribed verbatim by the researchers. Second, the Nvivo 10 software was used to code all transcripts line by line. Concepts that emerged from the interview data were identified as themes and organized to construct the results. Lastly, the researchers referred to existing literature to make sense of the findings. Several crucial statements from the interviewees were selected to exemplify the findings of the study. In order to fully protect and ensure the anonymity of the participants, their names and ages were concealed and showed as “Respondent XX (number)” in the results section. Notably, participants’ numbers were randomly identified.

## Results

### Addiction symptoms

This study used Brown’s criteria for technological addiction to analyze smartphone addiction symptoms among young Chinese working adults [22, 23]. Withdrawal, salience, and conflict with other activities were the typical smartphone addiction symptoms manifested by the respondents.

#### Withdrawal

Withdrawal refers to experiencing negative feelings when individuals are unable to engage in the activity [22, 23]. It was the most prominent symptom of smartphone addiction identified in this study because a number of young employees indicated that they felt anxious, uneasy, and even panicky when they were unable to use their smartphones. For example, one respondent whose job involved high mobility stated that:

“If I don’t see my phone for a while, I would panic. My work entails extensive traveling. In most situations, my smartphone is the only thing I can rely on to obtain information. When my phone has been out of range for an hour, I become very nervous and start wondering if my colleagues or parents are looking for me because of an emergency…” (Interviewee #30)

In particular, the coding results revealed three main situations where the young employees experienced withdrawal symptoms related to smartphone use. In the first situation, unpleasant feelings arise when they leave their phones at home. “Whenever I leave my smartphone at home, I go back to get it immediately without any hesitation. I still do so even when I might be late for work because of it. Otherwise, an anxious feeling would accompany me the whole day,” a respondent (Interviewee #14) said. In addition, several young employees would immediately apply remedial measures to reduce their negative feelings by seeking for alternative ways to inform others about how to reach them. A young worker stated that:

“I immediately use QQ and other social media channels to let my friends and colleagues know that I do not have my phone with me. I have to let them know how they can find me (e.g., via QQ and office land-line phone) in case an emergency occurs.” (Interviewee #32)

Withdrawal emotions also emerged when the participants’ smartphones ran out of battery. Several young employees recalled becoming very impatient in such situations. “If my phone ran out of battery, I would definitely freak out. Therefore, I charge it every night and never allow it to have less than 20% battery level,” a young respondent (Interviewee #25) said. Another employee, who appeared to be a heavy smartphone addict, indicated that phone battery levels under 40% are unacceptable to him because:

“If my phone’s battery level is lower than 40%, I would become very insecure and would have to charge my phone immediately. If I were outside, I would look for a public charging station to charge the phone. If no public charging station is available, I would start my car and use the automobile phone charger to have my phone charged as soon as possible.” (Interviewee #24)

To prevent the unpleasant feelings associated with flat battery levels, several employees even carried power banks and phone chargers with them wherever they went.

The young employees in this study also experienced undesirable feelings when their smartphones did not have any reception. One employee described the negative emotions he felt when his smartphone had no signal in several remote areas:

“At that time, I felt so bad…very nervous and restless… I kept restarting my phone because I thought it would help. I also constantly shook my phone in the hope of getting a signal. I felt like… my whole body was very uncomfortable.” (Interviewee #14)

One respondent (Interviewee #26) also reported that he often carried two SIM cards from two different telecom companies with him in order to avoid out-of-signal situations. “If one SIM card cannot get a signal in one place, I would change to the other one immediately. Otherwise, I would be very uneasy,” he said.

Notably, several respondents reported that to limit their unpleasant feeling, they carry their smartphones wherever they go. Even when they go to the bathroom, they carry their phones with them.

#### Salience

Salience represents the condition that an activity dominates people’s thoughts and behavior [23]. In this study, the salience symptom was mainly revealed as regularly using or checking smartphones while doing daily activities. The respondents admitted that they frequently checked their phones for work purposes. One respondent explained that:

“I check my phone every one or two minutes. I am afraid of missing any important messages from my customers. My job requires me to be contactable all the time.” (Interviewee #2)

Another young worker (Interviewee #23) admitted that checking his smartphone was a crucial part of his routine. “When I wake up in the morning, the first thing I do is check my phone. Every time I go out, the first thing I check is whether I have my phone with me. Even when I am having dinner, I also check my smartphone …,” he said.

Additionally, the coding results revealed that smartphones not only led to the young employees’ checking habits but also preoccupied their minds all the time. One interviewee (Interviewee #13) stated that even when he was doing other important tasks and his two hands were busy, he could not help wondering if new messages have come in.

#### Conflict

Conflict is the third smartphone addiction symptom identified in this study. According to Brown, conflict reflects how people’s activity leads to a clash with other people and other activities [23]. Throughout the interviews, the young employees narrated that their smartphone use interfered with various daily activities, such as family gatherings, social gatherings, and work.

Conflicts with family activities was the most frequently mentioned theme in the interviews. The respondents indicated that their smartphone use interfered with their family gatherings. “My mother often shouts to me, ‘Don’t play on your phone anymore!’ when we have dinner together,” a respondent (Interviewee #28) said. Another employee described his parents’ complaints about his smartphone use:

“When I am having dinner with my parents, I seldom talk to them. I just sit there, looking only at my smartphone and checking new messages or emails. My parents have complained about this many times…” (Interviewee #2)

Smartphone use had also interfered with the social gatherings of the respondents. The abovementioned respondent admitted that he was often criticized by his friends because of his smartphone use and said that “once when my friends and I had dinner together, I checked my phone status constantly and replied to messages. At that time, my friends criticized me and asked me to concentrate on having dinner with them.” Moreover, smartphone use had disrupted dating as well. Another interviewee said that using his smartphone for news updates had disrupted his romantic relationship:

“My girlfriend has complained about my smartphone use many times. When I am out on a date with her, she wants me to talk to her… but I always have phone calls and stare at the phone screen to check for messages or send emails. We have had many fights because of this…” (Interviewee #24)

With regard to smartphone use causing conflicts with work, a few respondents indicated that their smartphone use decreased their job efficiency because it seriously distracted them from the work they were doing. One respondent who needs to stay connected via his smartphone said:

“Previously, I could focus on one task in one hour. Now, I am unable to concentrate on one task within five minutes. I am easily distracted from the work that I am currently doing because I always think about my phone and keep checking it at work. If a new message comes in, I stop my current task and start dealing with this new message instead. It is like my attention is always distracted by my phone. Consequently, my job efficiency has decreased. Even worse, I sometimes make mistakes due to the distraction.” (Interviewee #13)

#### Phantom phone signals

Phantom phone signals (PPS) is a new symptom identified in this study. Similar to studies on mobile phones [30, 31], this study defines PPS as an individual’s illusory perception of a phone signal indicating an incoming call, message, or social media notification when no such signal is transmitted in reality. Twenty-one young employees reported that they have experienced phantom vibration or ringing when nobody has called or texted them. “Sometimes I think I hear my phone ring or vibrate. After checking it, I find that nothing has happened…my phone is completely still. This situation happens very often,” an employee (Interviewee #1) said. Likewise, another respondent who used a smartphone very frequently described a similar symptom:

“I think I really have to keep myself away from my smartphone sometimes. I am serious. I am now experiencing phantom vibration or ringing very frequently. I often feel that my phone is ringing or vibrating in my pocket, but the reality is it is not. Am I sick? It scares a little bit.” (Interviewee #14)

The respondents further explained that this PPS symptom often occurs when they are waiting for important messages or in public places, such as busy streets or crowed shopping malls. For example, an employee said:

“After I introduce the products, I normally need to wait for my customers’ replies about whether or not they would buy our products. While waiting, I check my phone repeatedly because I constantly feel that my phone is ringing or vibrating when in fact, there is no alert at all.” (Interviewee #19)

With regard to the PPS symptom that occurs in public places, an interviewee (Interviewee #26) shared his latest experience on the street. He said, “The last time I was walking down the street where loud music was being played, I thought my phone was ringing. I kept checking it again and again but found out that I was only imagining it.” Another example came from an iPhone user who has experienced phone ring hallucination frequently. He (Interviewee #30) mentioned that whenever he hears an iPhone ringtone nearby, his first reaction is to check his own phone to see if someone is calling him.

### Psychological factors

From the interviews, three psychological factors drawn from the Big Five personality traits were determined to affect young employees’ smartphone addiction; these three are conscientiousness, neuroticism, and extroversion.

#### Conscientiousness

Conscientiousness, which is characterized by achievement, self-discipline, and dutifulness, was the most mentioned psychological factor related to smartphone addiction among young Chinese workers. The results showed that conscientious persons, especially those who work in professions with demands to stay connected and accessible, are likely to develop smartphone addiction. One respondent (Interviewee #26) regarded himself as a dutiful person and said, “I cannot allow (work-related) problems to happen due to my own carelessness or irresponsibility. Thus, I make myself available 24 hours via my smartphone to stay connected with work.” He holds his smartphone at all times out of fear that he would miss important calls on weekends or holidays. Another respondent shared similar information regarding the relationship between conscientiousness and smartphone addiction by stating that:

“I am a conscientious person and sort of results-oriented. I am very aware that timely responses to my customers’ concerns are crucial for my work. My smartphone serves as a convenient platform to immediately understand customers’ needs and handle their matters. If I were to forget to bring my smartphone, I would be very nervous and unsettled because it might deteriorate my job performance…” (Interviewee #30)

#### Neuroticism

Neuroticism, which refers to a person’s tendency to be emotionally unstable, was identified as another psychological factor that affects smartphone addiction. According to the coding results, neurotic workers who become anxious easily and are sensitive to others’ responses are likely to develop smartphone addiction symptoms. One employee mentioned:

“I easily worry about many things, such as missing important calls, scheduling work wrongly, and insufficient preparation for my tasks. When I forget to bring my phone, I feel very anxious and unsettled. Even when my phone is with me, I still worry about missing calls frequently.” (Interviewee #18)

With regard to sensitivity towards others’ feedback or evaluation, a young employee(Interviewee #7), whose job required him to stay in touch, expressed that, “… after my colleagues complained about their loss of connection with me for a few times, I began to pay close attention to my smartphone status because I really care about how my friends look at me. I don’t want to be viewed as an unreliable person.”

#### Extroversion

Extroversion was also identified as a psychological factor that affects smartphone addiction. Several respondents indicated that extroverts, who are described as sociable and talkative, are likely to rely on smartphones because these devices provide a convenient platform for them to communicate with others and express their opinions or emotions. For instance, one interviewee (Interviewee #27) remarked that, “I like making friends and engaging in sociable activities. The social media apps on my smartphone enable me to socialize with others, such as my colleagues and clients, and freely express myself. I really enjoy my smartphone and have become attached to it.”

However, an interviewee provided a different explanation for the association between extroversion and smartphone addiction. He believed that introverts are likely to become hooked on smartphones because the mediated platform smartphones provide allows them to relax during social interaction. He stated that:

“Smartphones provide a relaxed platform for people such as myself who are a little introverted. I am often too shy to talk face to face. Through my smartphone, I feel comfortable and relaxed. It’s like…I turn into a different me, an extroverted person, in the smartphone platform. This might be the reason I am dependent on my smartphone.” (Interviewee #3)

## Discussion

The findings showed that young employees in China exhibit various smartphone addiction symptoms, including withdrawal, salience, conflict, and PPS. Three key psychological traits (i.e., conscientiousness, neuroticism, and extroversion) were found to influence work-related smartphone addiction.

Withdrawal was the most commonly identified smartphone addiction symptom in current study. This result is consistent with that of previous studies which have shown that experiencing unpleasant emotions when smartphones are inaccessible is a key indicator of smartphone addiction [4, 15]. The higher the level of negative feeling young working people have without their smartphones, the greater their addiction to their phones. Moreover, this study identified three situations wherein young employees experience withdrawal symptoms related to smartphone use; these are leaving smartphones at home, running out of battery, and loss of phone signal. These three situations can be used to improve the measurement of the withdrawal symptoms of smartphone addiction because most previous studies did not specifically consider them [4]. Furthermore, the identified preventive measures (e.g., charging smartphones every night and always keeping a portable power bank) for reducing withdrawal can be incorporated into the instrument for measuring the withdrawal symptoms of smartphone addiction. Given that the negative feelings resulting from being out of smartphone contact or being unable to use smartphones may cause psychiatric and psychological problems [32, 33], researchers and health professionals should pay more attention to these feelings.

Regarding the salience symptom, this study showed that smartphones not only dominate young employees’ behaviors by compelling them to keep checking their phones but also override their thought processes by making them constantly think about it. This finding supports Brown’s classification of salience, which includes behavioral and cognitive salience [22, 23]. It is also similar to the findings of previous studies. For example, Bian and Leung found that smartphone addicts often feel preoccupied with their smartphones even when they are not using them [4]. Lee et al. discovered that several people are compelled to check their smartphones [15]. In addition, the association between salience and conflict symptoms should be noted because the respondents reported that thinking about their smartphone messages at work and regularly checking their smartphone status disrupted their work and family/friendly gatherings. This result is comparable to those of prior research which found that students’ grades decrease because of over-reliance on smartphones [4, 15]. An effective strategy to control or reduce the salience symptom must be developed because conflicts with work activities may reduce employees’ work productivity and consequently cause an economic loss to companies. Additionally, the three main types of conflicts identified in the current study (i.e., disrupting family gatherings, disrupting friendly gatherings, and distracting attention from work) can be added to the instrument for comprehensively measuring the conflict symptom of smartphone addiction among working adults.

PPS is a new symptom identified in the present study. It is not included in Brown’s criteria of behavioral addiction mainly because Brown’s criteria are generally used for understanding the addiction symptoms of technologies, which do not include mobile ringtone functions (e.g., computers, games, and the Internet) [22, 23]. Over two-thirds of the young employees reported that they have experienced the PPS symptom. This symptom is likely related to the job roles of employees. In several fields, such as marketing, consultation, and media, smartphones are the most frequently used tool for work. In most situations, employees in these fields have to immediately attend to work matters after receiving an incoming call, message, or social media notification. After being in a ‘standby’ state for a long time, the strong psychological hint could induce PPS. Meanwhile, increasing numbers of people using similar ringtones could deteriorate the PPS symptom. Currently, mainland China’s smartphone market is dominated by Samsung (27.8%) and Apple (19.9%) [34]. Given that the ringtone for all iPhone or Samsung users is fixed by default, it is difficult for people to distinguish whose phone is ringing, especially in noisy public places. Considering that PPS is common among young workers, future research should pay attention to it and use diverse research methods to understand it.

Notably, this study did not find evidence on three symptoms from Brown [22, 23], namely, tolerance, euphoria, relapse and reinstatement. On one hand, this result is probably due to the specific smartphone usage patterns of our interviewees. For these young employees, work and social interaction are the main purposes of using smartphones. Symptoms, such as euphoria, did not emerge because these two kinds of smartphone usage are basically instrumental and may not produce a “buzz” or “high” feeling. Additionally, the majority of the respondents admitted that they are addicted to smartphones mostly because of their work and not for personal fun. Thus, young working adults’ smartphone addiction symptoms are primarily manifested as not being able to live without the device, always checking it, and always thinking about it. On the other hand, this result may be due to the sampling technique used in the present study. The participants were recruited via the authors’ social network by using a convenience sampling method. As a result, the smartphone addiction symptoms identified in this study cannot precisely represent the entire working population. Future studies using a random sampling method are required to further test the applicability of Brown’s addiction criteria [22, 23].

Moreover, conscientiousness was found to affect employees’ smartphone addiction. This factor has been seldom related to explaining smartphone addiction because prior research revealed that mobile phone addiction is not a function of conscientiousness [35, 36]. Being one of the first to identify this association, the current study suggests that future research should pay more attention to this factor to obtain a comprehensive understanding of mobile phone addiction. Furthermore, this study discovered that conscientious workers are likely to develop smartphone addiction, which is contradictory to existing technology addiction literature that showed that conscientiousness is negatively related to Internet addiction [37], Facebook addiction [38], and social media addiction [39]. This result can be explained by the specific group of people that this study investigated. Past research mainly targeted adolescents or university students. To them, new media gadgets are primarily used for games and socialization. Hence, less conscientious adolescents/students are likely to develop addiction to these gadgets because they provide opportunities to procrastinate, such as surfing the Internet, socializing with friends, watching videos, and playing games. However, for working adults, new media gadgets (e.g., smartphones) are mainly used as working tools. Conscientious workers who are diligent and responsible are likely to rely on smartphones to ensure accessibility at all times. Consequently, smartphone addiction is developed. Conscientiousness is a key variable in understanding the difference in technology addiction (e.g., smartphone addiction) between adolescents and working adults.

In line with previous mobile phone addiction research [40, 41], this study found that several extroverted young employees are likely to become addicted to smartphones probably because extroverted workers are sociable and usually have large social networks. Hence, they may find the use of smartphones to communicate with others appealing, especially when their job demands frequent communication. The findings of Lin et al. also support this explanation; the study showed that sociable young smartphone users tend to have a high level of smartphone dependence [5]. However, in the present study, an introverted technical staff felt addicted to smartphones because he used his smartphone as a mediated communication platform to make his social interactions with others easy. Future research should test and clarify the relationship between extroversion and smartphone addiction, especially in a work setting.

Additionally, neuroticism was found to affect young workers’ smartphone addiction in the current study. This finding is consistent with those of prior mobile phone addiction studies that found that neurotics are likely to develop mobile addiction symptoms because they are highly sensitive to others’ responses [42]. For young employees whose job is about relationship maintenance or customer service, their neurotic personality makes them likely to become addicted to smartphones.

## Conclusions

Several important theoretical and practical implications can be drawn from this study. First, this study contributes to the knowledge on smartphone addiction among young working adults. As one of the first to explore smartphone addiction symptoms among Chinese working adults and the psychological factors that affect such addiction, this study greatly enhances our understanding of the issue. Second, this study extends the range of symptoms of smartphone addiction. For instance, the results revealed that salient and conflict symptoms are related to each other and associated with addiction. Third, the symptoms identified in this study can help improve existing instruments for measuring smartphone addiction. For example, the PPS symptom and preventive measures (e.g., charging smartphones every night and always keeping a power bank) can be incorporated into smartphone addiction instruments. Lastly, in terms of theoretical implication, this study suggests that using an in-depth interview approach to explore an emerging phenomenon is essential. Without utilizing an interview method, we would not have known that conscientiousness has a significant and positive influence on smartphone addiction. Hence, future research on this emerging phenomenon can draw lessons from the present research and try to use multiple approaches to derive robust results. In practice, this study alerts employers to pay attention to the symptoms of smartphone addiction among young employees, especially those with certain personal traits. Considering that employees’ smartphone addiction may lead to serious negative psychological outcomes and decreased job performance [10], companies and employers should develop strategies to curb this addiction. Moreover, the smartphone addiction symptoms identified from this study can help companies pinpoint employees who are addicted to their smartphones so that they can provide timely guidance at the early stage of the problem.

However, we acknowledge several limitations of this study. First, the sample size was relatively small and not randomly selected, which might weaken the generalizability and validity of the results. Future studies can use the results as a basis to develop a large sample survey that focuses on smartphone addiction among Chinese working adults. Second, this study only examined young working adults. Given that older working people may also experience smartphone addiction symptoms, future research should consider examining smartphone addiction symptoms among older working adults to obtain a comprehensive understanding of the issue. Furthermore, instead of one-on-one interviews, diversified research methods, such as longitudinal qualitative research design, that can explore changes in smartphone addiction symptoms over time could be applied to further understand smartphone addiction among workers.

In summary, this study provided an in-depth investigation of the symptoms of smartphone addiction among young working adults in China and the psychological factors that affect such addiction. The results offer a solid foundation for future research to examine smartphone addiction among working adults in China and elsewhere.

## Data Availability

The datasets used and analyzed during the current study are available from the corresponding author on reasonable request.
